# Study on major legal issues and solutions in pandemic treaty negotiations

**DOI:** 10.3389/fpubh.2024.1413036

**Published:** 2024-09-20

**Authors:** Weiqi Chen

**Affiliations:** Law School, Xinjiang University, Urumqi, Xinjiang, China

**Keywords:** international health rule of law, pandemic treaty, the novel coronavirus, a community of human health, International Health Regulations

## Abstract

In December 2021, WHO’s 194 member states began reaching a consensus to start the process of drafting and negotiating a pandemic treaty under the WHO Act. Although there is already a PHEIC system to deal with sudden public health events such as pandemics, the system is not sufficient to deal with global pandemic events. The draft WHO Pandemic Agreement reflects the negotiating process until 24 May 2024. The negotiating team is faced with legal issues such as the treatment of the relationship between the pandemic treaty and the International Health Regulations, the determination of the contracting model, the attribution of the pandemic definition power and the construction of the dispute settlement mechanism. Through a study of the articles of the current draft and a comparative analysis with other treaties, this paper discusses the need to distinguish the functions of the pandemic treaty and the International Health Regulations (IHR), adopt a soft and hard contracting model, establish an open and transparent pandemic determination mechanism, reform the institutional functions of WHO, and establish an effective dispute settlement mechanism in order to solve the above problems. Ultimately, fairness and justice in international public health governance will be achieved.

## 1 Introduction

The draft WHO Pandemic Agreement reflects the negotiating process until 24 May 2024. A preliminary draft of 37 articles focused on the use of terminology, the principle of objectives, pandemic prevention and public health surveillance, a “One health” approach to prevention, preparedness and response to pandemics, preparedness and resilience of health systems was formed. Member States have reached preliminary agreement on some of the chapters, there is initial convergence on some elements, and there are many texts where there is no convergence and where there is disagreement. The draft is more like a partial complement to the missing elements of the International Health Regulations 2005. It is debatable how the draft should be viewed in relation to existing international treaties or regulations.

The new coronavirus has a strong ability to “mutate,” and the variant Omicron strain is highly infectious, and the number of confirmed cases and deaths of the new coronavirus continue to soar. By mid-2024, the World Health Organization has reported more than 600 million cases, and there are signs of a local rebound in some countries and regions (1). Mankind must be clearly aware that viruses know no borders, diseases know no nations, and it is urgent to strengthen international cooperation. The Framework is based on the WHO’s document, A Potential Framework Convention for Pandemic Preparedness and Response The formulation of the Convention refers to it as the “Pandemic (framework) Convention.” This paper focuses on the legal issues of the relationship between the pandemic treaty and the International Health Regulations, the determination of the contracting model, the ownership of the pandemic definition right and the construction of the dispute settlement mechanism. In order to solve the above problems, it is necessary to distinguish the functions of the pandemic Treaty and the International Health Regulations 2005, adopt a soft and hard contracting model, establish an open and transparent pandemic determination mechanism, reform the institutional functions of WHO, and establish an effective dispute settlement mechanism.

## 2 The necessity to establish a pandemic treaty

### 2.1 Deficiencies in the current international pandemic preparedness regime

The International Health Regulation (IHR) is the WHO’s foundational treaty in the field of global public health governance and epidemic response. In response to the spread of epidemics, the IHR first established the Public Health Emergency of International Concern (PHEIC) system in 2005. Article 1 of the IHR defines PHEIC as an unusual public health event that “constitutes an international spread” that “poses a public hazard to other countries in the international community” and that “requires a coordinated international response” (see Article 1 of the IHR). PHEIC includes “Rules for Notification and Publication of Epidemics,” “Rules for Surveillance of Epidemic Control,” and “Rules for Control of Epidemics” (2). First of all, the determination of PHEIC depends on the information provided by the member States. The information gathering phase adopts a “surveillance—notification” model, and the IHR requires Member States to assess the nature of the event according to the process in Annex 2: If an event that may constitute a PHEIC is consistent, the Member State focal point should notify WHO within 24 h of the assessment (see Article 6 of the IHR; Annex 1). Second, after the notification, member States should also continue to fulfill their surveillance obligations, including subsequent information sharing obligations, verification of information on public events, and Member States should provide the greatest possible support to WHO in coordinating national epidemic response (see IHR, Articles 7, 10, 15). After the PHEIC is determined, the epidemic control stage is reached, and WHO can issue interim recommendations to coordinate and unify health measures in various countries to reduce the drawbacks caused by the lack of coordination of emergency measures in various countries. However, the provisional proposal has no legal binding force and enforcement force, and has the nature of soft law.

First, the PHEIC system helps to remove cumbersome political considerations to some extent, and the WHO’s professional advice can also help coordinate national actions to deal with the pandemic. But unfortunately, PHEIC is only limited to non-medical health measures, and does not involve the key links of epidemic prevention and control—pandemic data sharing, vaccine drug research and development, which is particularly important for the prevention and control of pandemics. Second, although the IHR and the related PHEIC system stipulate the obligations of States, because the IHR does not set penalties for member states’ violations of obligations, the IHR lacks “compliance gravity” and the incentive for States to comply is insufficient (3). For example, in line with PHIEC, IHR set notification obligations, but because of the fear of stigmatization, most countries did not fulfill their timely notification obligations when the COVID-19 pandemic arrived, thus missing the best opportunity to build an information network. In addition, countries failed to take additional measures in accordance with the requirements of the IHR. Sixteen experts and scholars in the field of health published an article in the Lancet entitled “Do not violate the International Health Regulations during the Novel Coronavirus Epidemic,” and they agreed that the additional measures taken by many countries against China during the novel coronavirus epidemic actually violated the relevant obligations under the IHR (4). Although the WHO has repeatedly called for the removal of trade barriers to combat the epidemic, little has been achieved (5). Finally, the current pandemic preparedness regime lacks a dispute resolution mechanism, which makes it difficult to resolve disputes between member States regarding the implementation of obligations, the designation of PHEIC, and the determination of the legality of restrictive measures.

### 2.2 Efforts by the international community to facilitate negotiations on pandemic treaties

Due to the inadequate response of the existing system during the COVID-19 pandemic, on 1 December 2021, the 194 member States of the WHO began to reach a consensus to start the process of drafting and negotiating a pandemic treaty in accordance with the WHO Act. Until June 2024, the 77th World Health Assembly (WHA) in Geneva failed to agree on key provisions in the “pandemic pact,” which aims to allow countries to work together more effectively than they did when COVID-19 emerged. More than 2 years of negotiations on a global treaty have ended in limbo.

The WHO has been involved in similarly tense negotiations over revisions to its International Health Regulations, which call for a financing mechanism to help countries respond to pandemics and access medicines, allow the WHO to share information if countries do not cooperate, and authorize “pandemic emergency” alerts. This is a more urgent warning than the existing public health emergency of international concern. “The success of the amendments to the IHR shows that in our divided world, countries can still come together to accomplish common cause,” said Tedros Adhanom Ghebreyesus, WHO Director-General. An intergovernmental negotiating body will continue to meet to discuss the pandemic Agreement and has pledged to finalize a new version for member states to vote on at the World Health Assembly in 2025 at the latest. “Although the agreement is not yet finalized, the World Health Assembly has finalized the way forward,” Tedros said (6).

## 3 Major legal issues covered by the pandemic treaty

Although the negotiation of the pandemic treaty is still in the stage, combined with the shortcomings and deficiencies exposed by the PHEIC system above, it is necessary for countries to consider the following issues when drafting and negotiating the pandemic treaty, and respond to them in the contracting process. Among the many legal issues to be solved, this paper focuses on four aspects: the relationship between the pandemic treaty and the International Health Regulations, the mode of making the pandemic treaty, the attribution of the pandemic definition power, and the dispute settlement mechanism.

### 3.1 Analysis of the relationship between the pandemic treaties and the International Health Regulations

The IHR was born in 2005 out of the need for epidemic control, and can be traced back to the first quarantine regulations in Venice, Italy, in the 14th century to contain the plague pandemic. In 1851, the world’s first regional convention on International Public health was established. In 1951, the WHO World Health Assembly adopted the global Treaty on International Health, but the document limited the scope of epidemics to only six diseases, including relapses and plague (7). Changed to the International Health Regulations (IHR) in 1969, and modified and refined in 1969, 1973, and 1981, they are still limited in the range of infectious diseases they can deal with. With the development of world trade, new epidemics continue to break out, which is difficult for 1995 IHR to cope with. The spread of SARS in 2003 prompted a comprehensive revision of the IHR in 2005, with Article 2 of the 2005 version extending the scope of application to the prevention and control of all diseases. Looking back at the history of conclusion and modification, IHR has experienced a cycle of “breaking a contract—triggering a modification—breaking a contract again—triggering a modification again.” After the outbreak of the novel coronavirus epidemic, IHR fell into a state of “broken” again, and there is no shortage of voices in the academic community for the reconstruction and modification of IHR text. For example, Wei believes that it is necessary to modify the IHR text based on the risk framework (8). Considering that the origin of the IHR is international epidemic prevention and control, and that the legislative technology for modifying the IHR has matured several times, the cost of modifying the IHR seems to be less than that of re-establishing the Pandemic Treaty. The following chart shows the contents of the articles in the IHR (2005) to visually show where the corresponding chapters need to be amended (Table 1).

**Table 1:** Overview of the main elements of the International Health Regulations (2005).

Serial number	Contents	Articles of the International Health Regulations (2005)
1	General provisions (purpose, scope, principles, transparency, timeliness and non-discriminatory implementation of health measures; general requirements)	Articles 2, 3, 42, 44.1
2	National focal point and relevant competent authorities	Articles 4, 22, Annex 7.2 (f)
3	Global surveillance system for public health emergencies	Articles 5.1, 5.2, 6.1, 6.2, 7, 8, 9.2, 10.1, 10.2, 13.1, 19 (a), 20.1, 21, 46, Annex 1
4	Public health response	Articles 10.3, 12, 13.1, 13.4, 13.5, 15, 17, 18, 43, 46, 48, 49, Annex 1
5	Ports of entry (international ports, airports, land ports) and international cargo, container, container handling areas	Articles 19–23, Annex 1B, Articles 23.1 (b), 33–35, 41
6	Vehicles and operators of vehicles	Articles 24–29, 37–39, 41, 43, Annexes 3, 4, 5, 8, 9
7	Special terms for international travelers	Articles 23, 30–32, 35, 36, 40, 43 and 45, Annexes 6 and 7

According to WHO and United Nations (UN) documents, the international community would prefer to conclude “A Potential Framework Convention For Pandemic Preparedness And Response.” WHO Director-General Tedros Adhanom Ghebreyesus has said that “the Omicron virus shows that the existing system restricts other countries from warning of a world pandemic, so we need to reach a new pandemic agreement” (9). According to the WHO document, A Potential Framework Convention for Pandemic Preparedness and Response, The 2005 IHR merely provides a legal framework for pandemic preparedness, and to ensure that sovereign governments take coordinated and effective measures against pandemics, as well as sustained and adequate political investment at the national and international levels, WHO is considering the establishment of a comprehensive legal treaty for pandemic preparedness and response. The pandemic treaty aims to: (1) Build national, regional and global resilience to pandemics; (2) Mobilize all member States to undertake the necessary international cooperation for timely prevention, rapid detection and effective response to the pandemic; (3) Ensure the coordination and equity of national policies on prevention and epidemic control; (4) Strengthen WHO leadership, including pandemic preparedness and response (10). One of the purposes of the PHEIC regime is to coordinate the actions of member States in response to health emergencies, and the purpose of the pandemic treaty and the PHEIC regime appears to overlap. Therefore, the first question that must be addressed in concluding a pandemic treaty is what is the relationship between the pandemic treaty and the IHR? Can the purpose of the pandemic treaty be fulfilled by the PHEIC regime as defined by the IHR? On the other hand, in the face of the great crisis of a pandemic, should the original 2005 IHR text be revised as a matter of priority? Or a new treaty to deal specifically with the spread of pandemics? Should the IHR or the pandemic treaty take precedence in the pandemic response? The essence of this issue is to deal with the 2005 IHR and the scope of application of the pandemic treaty, and only by dealing with this relationship can we clarify the positioning of the pandemic treaty.

### 3.2 Modalities for making pandemic treaties

The World Health Assembly (WHA), the core body of the WHO, as the main health specialized agency of the United Nations, has a wide range of “legislative powers.” Constitution of the World Health Organization (WHO) Article 19 gives the WHA the power to conclude treaties or framework conventions (see article 19 of the Charter of the World Health Organization) and article 21 gives the WHA the power to conclude regulations in areas such as health and quarantine (see Charter of the World Health Organization, article 21). WHO has repeatedly stated that the main objectives of the Pandemic Treaty are to promote multilateral cooperation, strengthen the global capacity to respond to the pandemic, and enhance the sustainability, equity, participation and transparency of the response (11). In order to achieve the goal of preventing a pandemic, the negotiating team must consider what kind of contracting model should be adopted for the pandemic treaty. Is it feasible to adopt “hard law” as the basis for a pandemic treaty in order to overcome the shortcomings of soft law mentioned above?

The legislation in the field of international public health has certain particularity. Recalling the experience of the conclusion of the IHR, scholars pointed out that the IHR adopted a safety framework to coordinate the actions of member states in the field of public health (12). The so-called security framework is a framework set under the “security” theory proposed by the Copenhagen School (13), which believes that the mover who wants to formulate a norm first expresses the threat of a certain problem with language behavior, and declares that unconventional means must be taken to contain it. If the audience accepts this view and agrees to adopt legal regulation, the issue is said to have been secured (14). The security mechanism adopted by IHR is specifically reflected in that WHO, which has professional knowledge and epidemiological analysis ability, proves that a certain disease is “threatening” (for example, the disease poses a threat to people’s health and the security of the international community) based on scientific evidence, and indicates that relevant measures must be taken to resist the disease, so as to call on countries to comply with the pandemic norms (15). Within the framework of security, there is no concept of compulsory legal rights and obligations, and WHO uses its professional knowledge to provide medical advice to its member states, in exchange for part of the sovereignty of member states to the maximum extent—member states take the initiative to publicly report domestic emergencies to the international community and take corresponding health measures in accordance with the recommendations of WHO. While enhancing the transparency of the public health measures taken by countries, we will also ensure the coordination of the collective response to the epidemic from the perspective of common security. Under the framework of security, the legislation and law enforcement in the field of international health are mainly based on the recommendations, standards and guidelines issued by the WHO, which are generally not mandatory, and countries will not suffer any adverse consequences for violating the system. The lack of mechanisms to guide member states to comply with the treaty is criticized by most countries. However, in order to get countries to reach an agreement as quickly as possible, the IHR set up a lot of soft law and principle provisions. This is because it is not easy to strike a balance between the interests of countries in the field of public health. Public health involves the most sensitive topic of people’s security among national sovereignty. Soft law and principled provisions are less mandatory for countries and have greater flexibility, and member states generally do not pay too much “sovereign cost” to accept relevant systems. In addition, soft law provisions leave room for follow-up negotiations between countries, which is conducive to reaching a compromise between countries with different strengths, interests and values (16). If a convention with mandatory binding force is needed, it will take a longer negotiation time for the negotiating team, and it seems difficult to reach the goal of concluding the treaty by 2024, and the adoption of soft law or model law will help speed up the process of concluding the treaty to a certain extent. In addition, some scholars have pointed out that international soft law can serve as a model for international actors due to its good implementation effect, and at the same time provide guidance for the establishment of new laws (17). At the beginning of the formulation of some international soft law, the participants were only a few countries or international organizations, but if their advanced concepts and good operational effects are demonstrated in the law enforcement practice, other countries’ willingness to participate in the practice of soft law can be enhanced (18). However, as a result, the problem of more soft law than hard law in the field of public health mentioned above has not been solved, and the conclusion of the pandemic treaty seems to be in a dilemma. Thus, what model the pandemic treaty should adopt has become a question for the negotiating teams to consider.

### 3.3 Attribution of pandemic definition power

Who should define a pandemic? Do Member States have the autonomy to define a pandemic? Or will the definition of a pandemic be left entirely to the WHO?

The 2005 IHR provides for the final decision of WHO’s PHEIC. The decision of the WHO Director-General to identify a PHEIC relies on information communicated by Member States, which are required to notify WHO of an unusual public event in accordance with the four criteria set out in Annex B of IHR 2005. At this level, member States have a certain influence on the decision of PHEIC, but the WHO Director-General has the power to make a final decision on PHEIC. From the point of view of the procedure for determining PHEIC prescribed by the IHR, the Director General of course needs to consult with each Member State on whether it constitutes a PHEIC, but if the Director General and each Member State do not reach an agreement on whether it constitutes a PHEIC within 48 h, The Director-General may directly decide on a PHEIC (see IHR, Article 6, Article 12). Dozens of experts selected from a roster of experts form an emergency Committee (the Emergency Committee), which submits its views to the Director-General, who ultimately decides on a PHEIC (see IHR, Article 49). According to the IHR, the Director-General is required to consider three substantive elements: “Does it constitute an international spread,” “does it constitute an international public health risk” and “does it require a coordinated international response.” However, once these three elements are met, does it mean that the WHO Director-General must declare PHEIC? Does the Director-General have discretion and discretion in the determination of PHEIC? The definition of PHEIC seems to be clear, but every time PHEIC is applied in the specific process, there are quite complex implementation problems, causing countries and experts in related fields to pay attention to the level of global health governance. When influenza A (H1N1) began to spread in 2009, then WHO Director-General Margaret Chan identified it as PHEIC and warned it as the highest level of influenza pandemic—Level 6. However, H1N1 did not spread widely in the world, but this action caused worldwide panic (19). Some scholars have pointed out that the Director General of WHO directly identified the influenza circulating in North America as PHEIC may be motivated by the profit motive of related pharmaceutical companies (20). This decision-making error led to the WHO being overly nervous in identifying PHEics in the future. After the emergence of the Ebola virus in 2014, infected areas such as North Africa and Libya officially notified the WHO of the epidemic situation in March 2014, but it was not until August of the same year that the Director-General of WHO announced the formation of PHEIC. Since then, the Republic of Congo has repeatedly proposed to the WHO to recognize Ebola virus as PHEIC, but has been rejected. In 2018, the World Health Organization held a meeting and determined that the Ebola virus outbreak in the Republic of the Congo has not met the conditions to constitute PHEIC, because although the Ebola virus has spread to urban areas in the Republic of the Congo, health care workers have been disrupted, and there is a risk of further expansion of the epidemic, but considering the rapid and comprehensive response in the Republic of the Congo, The Director-General considered that the declaration of Ebola as a PHEIC had no added benefit but caused panic and unnecessary harm to trade and travel (21), so the WHO Director-General denied that Ebola constituted a PHEIC. Some scholars pointed out that the Director-General mainly considered the economic interests of Europe and its dependence on Guinea’s mining industry to not identify Ebola virus infection as PHEIC, indicating that the WHO Director-General did not fully judge PHEIC based on the three criteria under the 2005 IHR. The international community doubts that the WHO Director-General has a serious political bias when judging PHEIC. In July 2019, the Director-General finally determined that Ebola virus constituted PHEIC (22), but at this time, Ebola virus had spread rapidly and widely, and affected countries had missed the best time for epidemic prevention. Therefore, scholars constantly put forward the demand and voice of PHEIC transparency.

The nature of a pandemic is to some extent similar to PHEIC, so does the pandemic treaty still give the WHO Director-General, who has lost credibility to some extent, full power of definition? Do the Director-General’s procedures for defining a pandemic need to be improved? Can sovereign States have the right to define a pandemic on their own?

### 3.4 Establishing a dispute settlement mechanism for the pandemic treaty

As mentioned above, it is extremely unlikely that member states will reach an agreement to submit a dispute to arbitration after a dispute arises, and it is difficult for member states to reach an agreement to agree on arbitration in advance. WHO does not have its own specialized arbitration procedures and arbitration bodies. At the beginning of the pandemic treaty, there were many calls for the establishment of dispute settlement mechanisms in the field of public health.

Scholars pointed out that when WHO was established, there was no dispute settlement mechanism, and the implementation of treaties basically relied on the willingness of member states. This phenomenon was caused by historical conditions, not the fault of WHO, because the effectiveness of international law at that time depended on the consent of sovereign states (23). Based on this, the international community believes that the dispute settlement mechanism of WTO can be used in the field of public health, because the dispute settlement mechanism of WTO has formed a mature dispute research and judgment system and proved to have good dispute settlement effect. From the perspective of cost saving, WHO can use the dispute settlement mechanism of WTO to achieve dispute settlement. However, such an approach inevitably faces several problems: First, the WTO dispute mechanism is cumbersome and complex, which requires the parties to invest more time and money. Judging from previous WTO litigation experience, the expert panel takes an average of 12–16 months to complete the proceedings, and due to the outbreak and interference from the United States, the WTO Appellate body has been suspended since 2019. Fast-moving epidemics seriously affect people’s health, and lengthy dispute settlement mechanisms are powerless to stop the spread of epidemics. Second, the WTO’s dispute settlement system deals only with trade measures and does not cover all obligations in the field of international health. Whether countries are fulfilling their obligations under the IHR, whether national quarantine measures on population and travel are excessively restrictive, and whether PHEIC is reasonable as determined by the WHO Director-General cannot be considered by the WTO. The “international standards, guidelines or recommendations” set out in Annex 1 of the SPS (Agreement on the Application of Sanitary and Phytosanitary Measures) (24) does not include WHO recommendations. Therefore, in the WTO dispute settlement mechanism, the recommendations of WHO and the relevant laws under the WHO cannot be directly applied as legal provisions, and the WTO Appellate Body cannot directly review the legal provisions of WHO (25). Finally, the WTO dispute settlement mechanism does not aim to make up for the loss, but only corrects the improper interim measures of the accused country, and the loss of the country subjected to the improper measures cannot be compensated (26). Moreover, relying on the WTO’s study and judgment means that the settlement of disputes in the field of international health is entirely dependent on the judgment of other international organizations, and the autonomy of WHO as an international organization will be completely lost. To sum up, the dispute settlement mechanism of WTO cannot be directly applied to the dispute settlement in the field of international public health, and WHO needs a set of dispute settlement system applicable to the field of health. How to establish such a mechanism is a question that WHO must consider when making a pandemic treaty.

## 4 Recommendations to address the main legal issues of the pandemic treaty

In view of the above mentioned four aspects of legal problems, this article in the following part on these four aspects of the problem proposed solutions.

### 4.1 Distinguishing between the functions of the pandemic treaties and the International Health Regulations

The purpose of the pandemic treaty is to provide unified guidance to member States for timely surveillance and response to diseases that may develop into global pandemics. The outbreak of the novel coronavirus has exposed the disadvantages of fragmented response among countries, and the wavering epidemic prevention policies of Western countries have laid hidden dangers for the response (27). “We need to create an environment where every scientist, health worker and government can come together for a common cause,” said Charles, president of the World Health Summit of the Council of Europe. Working together to build new solutions to protect what is most precious to us—our health and lives (28). The pandemic treaty creates an environment of solidarity and cooperation in the face of an emergent pandemic.

In discriminating between the functions of the IHR and the pandemic treaty, we must return to the terms of reference defined in Article 2 of the IHR. Article 2 of the IHR states that its purpose is to provide relatively coordinated health measures while balancing international traffic and trade in response to public hazards. Although the IHR has been modified frequently, it has not been able to promote the adoption of relatively consistent border quarantine policies and measures in the event of an outbreak. Compared with emergency control, IHR puts more emphasis on risk management, emphasizing that member states should build core capacities in peacetime to ensure timely epidemiological monitoring and risk mitigation. The obligations set by IHR are more penetrated into the daily public health management of countries (29), and the pandemic treaty needs to make faster and more scientific responses to sudden diseases. Although the pandemic treaty and PHEIC overlap, their focus is different. According to the definition of PHEIC, only epidemics that have constituted a realistic “international epidemic” can be declared PHEIC, however, the pandemic crisis may not only include diseases that have constituted a realistic “international spread,” but also the “potential spread” epidemic sources need to be paid attention to, such as the previous Ebola virus. When the WHO initially refused to identify the Ebola virus as PHEIC, some scientists in the European Union pointed out that although the virus did not constitute an international spread for the time being, it appears that it is likely to constitute a potential spread from the infertility of the virus, and the eventual spread of the Ebola virus in Congo and other places also confirmed their concerns. Therefore, the pandemic treaty should not only target the diseases that have already constituted an international epidemic, but also prevent the diseases with epidemic potential, and give full play to the function and value of the pandemic treaty in detecting new epidemics. Pandemics should be purely a matter of scientific fact, requiring expert committees to conduct scientific analysis on the infectivity, pathogenicity and fatality rate of the infectious source, and determine whether a particular disease belongs to the category of a global pandemic, without considering factors unrelated to epidemics such as international traffic like PHEIC. The pandemic treaty focuses on the surveillance needs of pandemics, and the WHO should establish more laboratories around the world to track and trace the source, strengthening countries’ core capacity to respond to pandemics.

In addition, pandemic treaties need to help countries detect and prevent pandemics early and strengthen their resilience to future pandemics. More importantly, a pandemic treaty needs to address how to ensure equitable global distribution of vaccines and medicines, as well as the flow of pandemic data (30). Vaccines and drugs are the key to ending pandemics. The end of numerous pandemics such as smallpox in history is nothing more than the development of corresponding vaccines and drugs by human wisdom, and how to make the distribution of vaccines and drugs equitable around the world is a major issue that needs to be considered in the pandemic treaty. Pandemics, by their nature, can only be based on population big data studies, not individual clinical trials. This means that the sharing of information and data between the pandemic country and other countries is the basis of all pandemic research, and how to ensure the flow of data from each country without endangering the data security of that country is also an issue that needs to be considered by the pandemic treaty, which is not covered by the IHR.

There is no complete separation between the pandemic treaty and the IHR, and the establishment of the global pandemic early warning system cannot be separated from the completion of the core capacity building requirements of the member States under the IHR, and timely and effective notification of epidemic information within sovereign States. Pandemic prevention is also one of the core functions of the IHR, which also assumes the responsibility for disease prevention and control in addition to pandemics, as well as the requirements for the physical and mental health of the citizens of member States. The pandemic treaty and the IHR form a closed loop for public health governance and reduce governance vacuums.

### 4.2 Adopt both the soft and hard contracting model and introduce the concept of “human health community”

In this paper, it is expected that the conclusion of the pandemic treaty will be concluded as soon as possible, and the “Framework Convention + Protocol” model will be adopted. Provisions of a soft law nature were desirable for the framework Convention, while more provisions of a hard law nature, such as specific quarantine measures, could be included in the protocol. At the same time, the IHR should be amended to eliminate the ambiguous provisions in it, and the dispute settlement mechanism of WHO should be constructed to make it have “teeth,” and the mechanism to cooperate with the IHR and the pandemic treaty should be established.

It is not realistic to completely “legalize” public health. However, even for soft law, the introduction of good ethical values in the process of the creation of soft law can also help to form the awareness of resource compliance of international actors and break through the barriers of state-centralism (31). Introducing the values of a community with a shared future for mankind through extensive consultation, joint contribution and shared benefits will help build and guide countries to keep their commitments. When the value concept of “the earth is a community with a shared future that shares weal and woe” goes deep into the value concept of every sovereign state and social organization, countries will be more inclined to abide by the provisions of the Convention and fight the epidemic together under the leadership of this value. Taking into account the discussion earlier in this article on the need to distinguish between the pandemic treaty and the IHR, adopting the Framework Convention + Protocol format would not only provide a clear list of the priorities of the pandemic treaty, but also allow member States to choose whether to join the subsequent protocol, which would speed up the conclusion of the pandemic treaty. After countries have reached a broad consensus on the basic principles in the framework Convention, with the in-depth development of practice, specific procedures can be refined in the protocol (32). Measures of a procedural and legal nature could be included in the protocol, such as pandemic notification procedures, exchange and disclosure of epidemiological data, and the transfer of technical patents for vaccines and drugs. Since the above issues involve relatively sensitive internal affairs of States, States are allowed to make certain reservations to the Protocol while acceding to the framework treaty. The pandemic treaty establishes, as soon as possible, convention bodies such as the Conference of the Parties to the Convention to hold countries accountable for fulfilling their obligations under the pandemic treaty. The Conference of the Parties is conducive to the establishment of a global epidemic reporting system, which helps WHO to summarize data with a problem-oriented approach and identify the shortcomings of member states in the implementation process (33). The provision of a special fund under the pandemic treaty for core capacity building should be linked to countries’ compliance. Some scholars have pointed out that linking the issuance of special funds to the implementation of the convention can not only eliminate the economic worries of developing countries in building core capacity, but also urge countries to implement the convention (34).

The IHR has been concluded for a long time and has gained considerable authority among the member states. Therefore, it is appropriate to codify the provisions in the IHR to set mandatory rights and obligations for member States, and this change will also facilitate the response to the pandemic in line with the pandemic treaty.

### 4.3 Establish an open and transparent pandemic definition mechanism led by expert committees

The IHR delegated to WHO the authority to determine PHEICS, with the aim of alerting the world to unusual events, while adhering to the principle of transparency. Pandemic and PHEIC are not controlled by a single sovereign state, which is doomed to define the pandemic from the perspective of a single country will have limitations, so the author opposes the definition of a single member state. The right to define pandemic should still be in the hands of WHO with professional knowledge, and recognizing WHO’s right to define is also recognizing WHO’s right to information governance (35). However, the establishment of epidemic information network and the exercise of WHO’s power to define epidemic diseases cannot be separated from the cooperation of member states. In order to establish an adequate and effective emergency response mechanism, timely and effective notification by sovereign states must be relied on. This does not deny the value of the rapid identification, notification and response norms established by PHEIC just because a country has failed to comply with its notification obligations (36). The first step in identifying a pandemic is the same as identifying a PHEIC, and WHO relies on notifications from sovereign states as a basis for decision-making. However, the author believes that sovereign countries should be given some discretion in the process of pandemic notification, that is, sovereign countries have the right to determine the nature of the epidemic and whether it constitutes a pandemic according to their domestic situation, and then decide whether to notify, rather than forcing sovereign countries to report all the epidemics in their country.

Specialized expert committees should be formed under the pandemic treaty, Expert committees shall be established by the WHA in accordance with the Regulations on Advisory panels and Expert committees of the World Health Organization The Organization selects and sets a certain term and change system. As mentioned above, the determination of a pandemic should be a purely scientific matter, and in order to ensure the scientific and accurate decision-making, the definition of a pandemic should be decided collectively by experts with professional knowledge, so the final right to define a pandemic should be delegated to the expert committee. The author believes that the list of experts should be made public when defining a pandemic, because compared with government organizations such as the Centers for Disease Control and Prevention authorized by sovereign states to deploy medical resources, WHO can dispatch resources are limited, and credibility is the most important resource for WHO. Open rosters of experts contribute to the establishment of a transparent pandemic identification mechanism, which is the premise and foundation of WHO’s global role. But to address the fear that experts will be persecuted for making pandemic decisions, the WHO should play its protective role and establish a system of relevant exemptions. As long as an expert determines the nature of a pandemic on the basis of his or her expertise, even if it does not eventually spread worldwide or is caused by force majeure such as accidental mutation of the virus, the expert should be exempted from the corresponding responsibility. Expert committees should collectively decide on pandemics and be collectively responsible for the declaration of pandemics. Rather than the WHO Director-General deciding on a PHEIC alone, a collective definition of a pandemic can help bring together collective wisdom, and establishing collective accountability can also help reduce the fear of individual decision-making.

### 4.4 Reform the institutional functions of the WHO and establish an effective dispute settlement mechanism

In order to improve global health coordination and fair handling of international health governance disputes, in the preliminary draft agreed in May 2024, the issue of dispute settlement is included in article 25, which reads as follows: “In the event of a dispute between two or more Parties regarding the interpretation or application of the WHO Pandemic Agreement, the Parties concerned shall seek to resolve the dispute through negotiation or any other peaceful means of their own choice, including good offices, mediation or conciliation, through diplomatic channels.” If no solution is reached by such means, the Parties concerned may, with their written consent, resort to arbitration in accordance with the PCA Arbitration Rules 2012 or subsequent rules, unless otherwise agreed by the Parties to the dispute.” This article holds that such dispute settlement provisions are too broad or the application is not specific and targeted.

WHO defines itself as a professional and guiding organization. For a long time, its main functions have been focused on the establishment of professional standards, the formulation of disease prevention and control plans, the assistance of member states to strengthen core capacity building and the response to public health crises (37), but it lacks the capacity of law enforcement and supervision. Therefore, if we want to establish a dispute settlement mechanism, first of all, we should gradually transform the functions of WHO and empower WHO. The idea of global governance, led by the US, permeates the WHO; The function of WHO mainly depends on the recognition of major countries and their response to the quarantine of WHO in key domestic policy areas (38), and over-relies on the implementation of treaties by member states, which results in the situation that the functions of WHO are constantly diluted in the game between major powers. Financial constraints make it harder for the WHO to fulfill its enforcement role. Therefore, this paper holds that WHO should attract extensive financial support and seek cooperation with individual funds and non-governmental organizations, so that WHO can have a greater say, and sufficient funds are conducive to WHO’s establishment of its own arbitration institution. In the May 2024 draft treaty negotiations, article 20 on “sustainable financing” went further: “Parties shall, in an inclusive and transparent manner, provide, to the extent practicable, more sustainable and predictable financing for the implementation of this Agreement and the International Health Regulations (2005)”; “Promote, as appropriate, innovative financing measures within relevant bilateral, regional and/or multilateral financing mechanisms, including the reformulation of transparent financial plans for pandemic prevention, preparedness and response, in particular for developing country Parties facing financial difficulties”; And “encourage inclusive and accountable governance and operational models of existing funding entities to minimize the burden on countries, broadly improve efficiency and coherence, increase transparency, and respond to the needs and national priorities of developing countries.” There are not many differences among the States parties in these areas, and the consensus has been reached, which has laid the foundation for the operational implementation of the sustainable financing program in the future.

With respect to dispute settlement, the WHO arbitration body should establish different procedures for different matters: In cases of disputes between rights and obligations between two States, such as disputes between one State over the timely compliance of notification obligations by the other State and additional measures imposed, the arbitral body shall be a neutral arbiter and, to the extent possible, make decisions on measures related to the pandemic in accordance with existing scientific summary procedures. For member states that fail to comply with the ruling, “reputational punishment” sanctions mechanisms such as notification to the international community can be adopted; for the determination of a pandemic or PHEIC, there is no neutral status of the arbitral tribunal. At this time, WHO accepts the supervision of each member state and responds to the questions of member States in a timely manner. Because of the rapid spread of the pandemic and the need to focus on timeliness, WHO must establish a rapid and efficient response mechanism. According to Article 56 of the previous IHR, if the member states have objections to the identification of PHEIC, they must pass the “vote” of the health Assembly in order to get a response, but the identification of the pandemic should adhere to the concept of science, rather than relying on the “vote” influenced by political factors, so that the member States directly to the expert Committee is more conducive to the resolution of disputes. The arbitral institution also has the obligation to respond to disputes arising from the definition of a pandemic in the Special Committee, and timely response to the definition of a pandemic will also help improve the transparency of the definition of a pandemic.

The establishment of an effective dispute settlement mechanism is inseparable from the cooperation between international organizations. Facts have proved that although the WTO dispute settlement mechanism does not fully meet the needs of international health governance, the effectiveness of its joint governance with the WHO cannot be ignored. Mutual cooperation is also conducive to managing the problems of multi-headed and unconcentrated power, forming complementary advantages (39). This paper holds that the greatest advantage of WHO’s cooperation with WTO lies in promoting countries’ compliance with the WTO’s current effective dispute settlement mechanism. The reason is that different from the public health field, which presents indirect potential benefits, countries tend to pay attention to the economic field with direct interests, and the influence of WTO is more conducive to promoting member countries’ compliance with the WTO. Global governance to control pandemics requires additional measures on trade, and the trade order is controlled by additional measures on international health. Cooperation between the WHO and the WTO is also conducive to resolving disputes arising at the intersection of international health and trade.

## 5 Conclusion

The process of negotiating the pandemic treaty may have been lengthy, but it filled a gap in the previous epidemic response regime. Although the establishment of a convention cannot avoid facing various problems during the negotiation process, the author believes that a good system will certainly benefit mankind. In the process of the negotiation of the pandemic treaty, clear positioning of the pandemic treaty, clear division of labor between the pandemic treaty and IHR, and prevent the vacuum of international health governance should be the primary issue to be solved; Adopting the form of “Framework Convention + Protocol,” the model of both hard law and soft law and introducing the value concept of the community of human destiny will help accelerate the signing of the treaty and establish a compliance mechanism based on common values, and form a consensus of consciousness and cognition. Moreover, there are still many loopholes in the chapters of the draft pandemic treaty on “pathogen acquisition and benefit-sharing system,” “supply chain and logistics,” “procurement and distribution,” and “sustainable financing,” which need to be further supplemented. The specific improvement of the draft and the problems encountered in the follow-up implementation are areas that scholars should continue to pay attention to. As discussed in the article, the establishment of an open and transparent pandemic identification mechanism led by expert committees will not only contribute to the prevention and control of pandemics, but also contribute to the building of the reputation of WHO. Finally, the reform of WHO’s institutional functions and the establishment of an effective dispute settlement mechanism to resolve disputes are conducive to promoting the compliance of member states, preventing countries from abusing health measures to carry out trade protection in disguise, and helping to balance epidemic prevention and control and global trade. The COVID-19 pandemic is both a challenge and an opportunity, and a treaty to guide the world’s response to pandemics would lay a solid institutional foundation for future responses to public health crises. It is hoped that substantive progress will be made in the upcoming pandemic treaty negotiations.

## Data Availability

The original contributions presented in the study are included in the article/supplementary material, further inquiries can be directed to the corresponding author.
